# Somatic Tumor Mutations Detected by Targeted Next Generation Sequencing in Minute Amounts of Serum-Derived Cell-Free DNA

**DOI:** 10.1038/s41598-017-02388-7

**Published:** 2017-05-18

**Authors:** Marjolein J. A. Weerts, Ronald van Marion, Jean C. A. Helmijr, Corine M. Beaufort, Niels M. G. Krol, Anita M. A. C. Trapman-Jansen, Winand N. M. Dinjens, Stefan Sleijfer, Maurice P. H. M. Jansen, John W. M. Martens

**Affiliations:** 1 Erasmus MC Cancer Institute, Department of Medical Oncology and Cancer Genomics Netherlands, Rotterdam, The Netherlands; 2 Erasmus MC Cancer Institute, Department of Pathology, Rotterdam, The Netherlands; 3 Erasmus MC, Cancer Computational Biology Center, Rotterdam, The Netherlands

## Abstract

The use of blood-circulating cell-free DNA (cfDNA) as ‘liquid-biopsy’ is explored worldwide, with hopes for its potential in providing prognostic or predictive information in cancer treatment. In exploring cfDNA, valuable repositories are biobanks containing material collected over time, however these retrospective cohorts have restrictive resources. In this study, we aimed to detect tumor-specific mutations in only minute amounts of serum-derived cfDNA by using a targeted next generation sequencing (NGS) approach. In a retrospective cohort of ten metastatic breast cancer patients, we profiled DNA from primary tumor tissue (frozen), tumor-adjacent normal tissue (formalin-fixed paraffin embedded), and three consecutive serum samples (frozen). Our presented workflow includes comparisons with matched normal DNA or *in silico* reference DNA to discriminate germline from somatic variants, validation of variants through the detection in at least two DNA samples of an individual, and the use of public databases on variants. By our workflow, we were able to detect a total of four variants traceable as circulating tumor DNA (ctDNA) in the sera of three of the ten patients.

## Introduction

Blood-circulating nucleic acids are extracellular (cell-free, cf) residing DNA or RNA molecules (cfDNA or cfRNA) that likely originate from apoptotic or necrotic cells, or are actively released^1^. In cancer patients, cfDNA may harbor somatically derived tumor-specific mutations reflecting the genomic characteristics of an individual’s cancer, such as single nucleotide variants or structural rearrangements^2, 3^. The use of cfDNA as a so-called ‘liquid-biopsy’ is being explored worldwide, with hopes for its potential in providing prognostic or predictive information. In exploring the potential of cfDNA in oncology, valuable repositories are biobanks containing material collected over time. However, these retrospective cohorts have restrictive resources, including suboptimal specimen preservations such as formalin-fixed and paraffin embedded (FFPE) specimens or limiting amounts of DNA available for profiling, challenging the analysis of these cohorts.

In this study, we aimed to detect tumor-specific mutations in only minute amounts of serum-derived cfDNA. We present a NGS workflow using a custom 45-gene sequencing panel on the Ion PGM system applied to a retrospective cohort of ten metastatic breast cancer patients. This workflow includes comparisons with FFPE matched normal DNA from tumor-adjacent histologically normal mammary epithelium or *in silico* reference DNA to discriminate germline from somatic variants. To evaluate the detection of variants in minute quantities of DNA, we initially compared for two patients the performance of our method on standard amounts of primary tumor DNA with minute counterparts. Finally, for all ten patients, we identified variants in their primary tumor DNA (standard DNA quantities) and three consecutive serum samples (minute DNA quantities).

## Results

### Performance of The Custom Amplification-Based Targeted NGS Panel

Using our custom panel we sequenced a total of forty-six DNA samples, derived from fresh frozen (FF) primary breast carcinoma specimens (tumor), FF serum (cfDNA) and FFPE tumor-adjacent normal mammary epithelial specimens (matched normal). Primary tumor (n = 10) and matched normal (n = 6) DNA samples were analyzed using standard amounts of DNA input. The cfDNA samples (n = 30) were analyzed using minute amounts of DNA input (median 387 pg, interquartile range IQR 265–445 pg) as well as two replicates of primary tumor DNA for two patients (n = 4) (250 pg).

For performance assessment, we analyzed the following parameters of the data. Before mapping we analyzed 1) read length distribution and 2) per sequence GC content of the generated reads. After mapping against the reference genome we analyzed 3) read for each amplicon, 4) percentage of reads mapped to the targeted regions relative to all mapped reads and 5) read depth of those mapped reads. Finally, after calling single nucleotide variants deviant from the reference genome we analyzed 6) the percentage of bases called at the required read depth.

First, the distribution in read length of the reads of each sample was compared to the expected distribution in read length based on amplicon size of the panel (Supplementary Figure S1A). The highest peak of read length was at the expected 120 base pairs (bp) for the primary tumor DNA sequenced at standard and at minute amounts, as well as for the cfDNA sequenced at minute amounts, whereas the matched normal DNA had its highest peak at 90 bp (Kruskal Wallis P < 0.001). Second, we analyzed the GC content of the reads for each sample (Supplementary Figure S1B). The highest peak for GC content was at the expected 50% for the primary tumor DNA sequenced at standard and minute amounts, and the cfDNA sequenced at minute amounts, whereas the matched normal DNA had the highest peak at 40% (Kruskal Wallis P = 0.002). Third, we analyzed the amplicon performance for each sample type by the median reads per amplicon. The required read depth of at least 20x was obtained for 3019/3106 (97.2%) amplicons in matched normal DNA. The required read depth of at least 100x was obtained for 2973/3106 (95.7%) in the primary tumor DNA at standard amounts, 2953/3106 (95.1%) at minute amounts, and 2947/3106 (94.9%) for cfDNA at minute amounts. A total of 66/3106 amplicons (2.1%) did not reach the required read depths in all four sample types covering regions in 25 genes. Fourth, we determined the percentage of reads mapped to the targeted regions relative to all mapped reads. This percentage was for matched normal DNA sequenced at standard amounts (90.4%, IQR 76.2–93.1%) approximately 5% less compared to primary tumor DNA sequenced at standard amounts (95.7%, IQR 95.6–95.8%) or cfDNA sequenced at minute amounts (94.5%, IQR 94.3–94.7%) (Mann Whitney both P < 0.001). This percentage was for cfDNA approximately 1% less than primary tumor DNA sequenced at standard amounts (Mann Whitney P < 0.001). The percentage of mapped reads at the targeted regions did not differ between primary tumor DNA sequenced at minute (95.2%, IQR 95.2–95.3%) and standard amounts (Mann Whitney P = 0.8). Fifth, the median read depth of matched normal DNA (351x, IQR 139–756x) was lower than that of either primary tumor DNA sequenced at standard amounts (899x, IQR 527–1377x) or cfDNA sequenced at minute amounts (891x, IQR 530–1385x) (Mann Whitney P = 0.001 and P < 0.001). The median read depth of either cfDNA or primary tumor DNA sequenced at minute amounts was comparable to primary tumor DNA sequenced at standard amounts (Mann Whitney P = 0.6 and P = 0.1, respectively). Finally, after mapping of the sequencing reads, we called single nucleotide variants deviant from the reference genome to reveal germline single nucleotide polymorphisms (SNPs) and somatically acquired tumor-specific variants. Of the called variants, the variant frequency was calculated as the fraction of variant reads over the total reads at that genomic position. Based on our criterion of 10 or more variant reads, matched normal material – intended to detect germline SNPs at 50% or 100% variant frequency – required a read depth of at least 20x. The percentage of bases at a depth of at least 20x was median 94.6% (IQR 91.8%-97.1%) in the matched normal DNA. Other material – intended to detect tumor specific somatic variants – required a read depth of at least 100x. In the primary tumor DNA and cfDNA the percentage of bases at a depth of at least 100x was respectively median 95.4% (IQR 95.0%-95.9%) and 95.0% (IQR 93.8%-95.8%).

Taken together, these findings imply that DNA sequenced at standard or at minute amounts results in comparable amounts and quality of data, but that sequencing material derived from FFPE preserved specimens – as is our matched normal material – resulted in less read depth and biased data with respect to length and GC content of the reads.

### Defining the Somatic Origin of Variants

**Figure 1:**
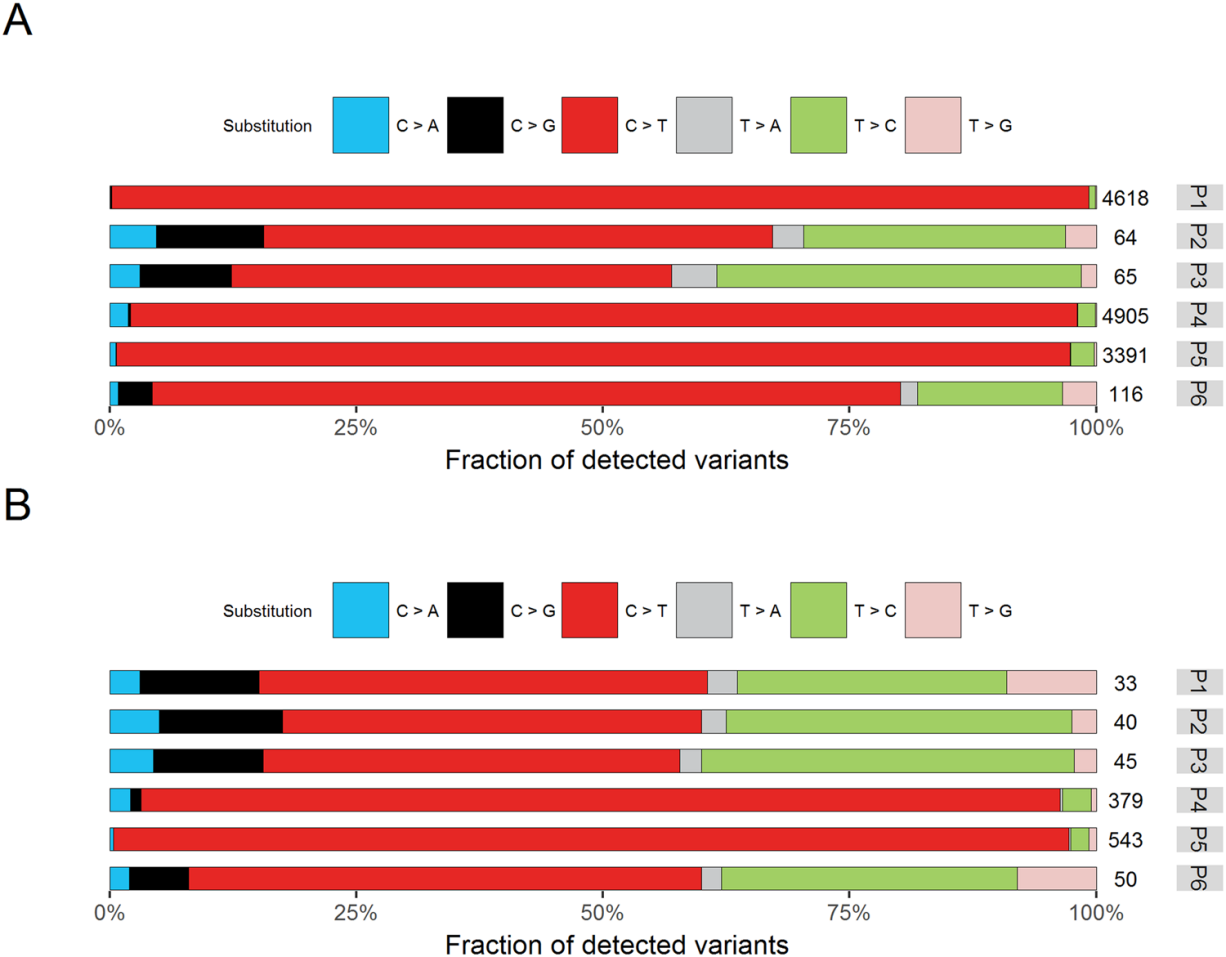
Substitution spectra of variants called relative to the reference genome in DNA derived from formalin fixed paraffin embedded (FFPE) matched normal specimens. (**A**) The contribution of the six possible base substitutions (C > A in blue, C > G in black, C > T in red, T > A in grey, T > C in green and T > G in pink) are depicted for each of six patients (P1 to P6) relative to the total variants in that sample. The total number of detected variants is depicted at the right end of the bars. (**B**) A threshold removing variants with variant frequencies below 35% was applied to FFPE preserved matched normal specimens. The resulting substitution spectra (as in **A**) are depicted for each of the six patients (P1 to P6).

To discriminate between SNPs and somatic variants in the primary tumor and cfDNA, we had FFPE-preserved tumor-adjacent normal mammary epithelium available for six of the ten patients. After calling single nucleotide variants deviant from the reference genome, an unexpected high and variable number of variants was detected in the matched normal DNA: a median of 1754 variants (IQR 78–4311 variants), as opposed to a median of 50 variants (IQR 46–54 variants) in the six corresponding primary tumor DNAs. The variants detected in matched normal DNA showed an enrichment for C > T transitions in their substitution spectrum, comprising median 86.0% (IQR 57.7–96.5%) of the total detected variants within a sample (Fig. 1A, red). This is different than the distribution for SNPs in the human exome (i.e. 6.7% C > A, 8.9% C > G, 52.2% C > T, 4.4% T > A, 24.4% T > C and 3.5% T > G^4^) or that of the FF primary tumor material (Supplementary Figure S2B). Variants in matched normal DNA are only expected to represent hetero- and homozygous SNPs at variant frequencies of 50% and 100% respectively. The C > T transitions in these FFPE specimens often had variant frequency far below 50% in most samples (Supplementary Figure S2A). Therefore, we explored the effect of excluding from our matched normal FFPE-derived DNA all called variants which had variant frequencies below expected frequency for heterozygosity. Exclusion of variants below 35% variant frequency in these samples greatly reduced the number of reported variants to a median of 48 (IQR 41–297 variants) per sample. However, for P4 and P5, the number of variants detected remained unacceptable high after exclusion of variants below 35% variant frequency (resp. 379 and 543, Fig. 1B) prompting us to omit all the data of those two matched normal samples for discrimination between germline SNPs and somatic tumor variants. For the other four patients, the variants detected in matched normal DNA after ≤35% variant frequency curations were considered germline SNPs.

**Figure 2:**
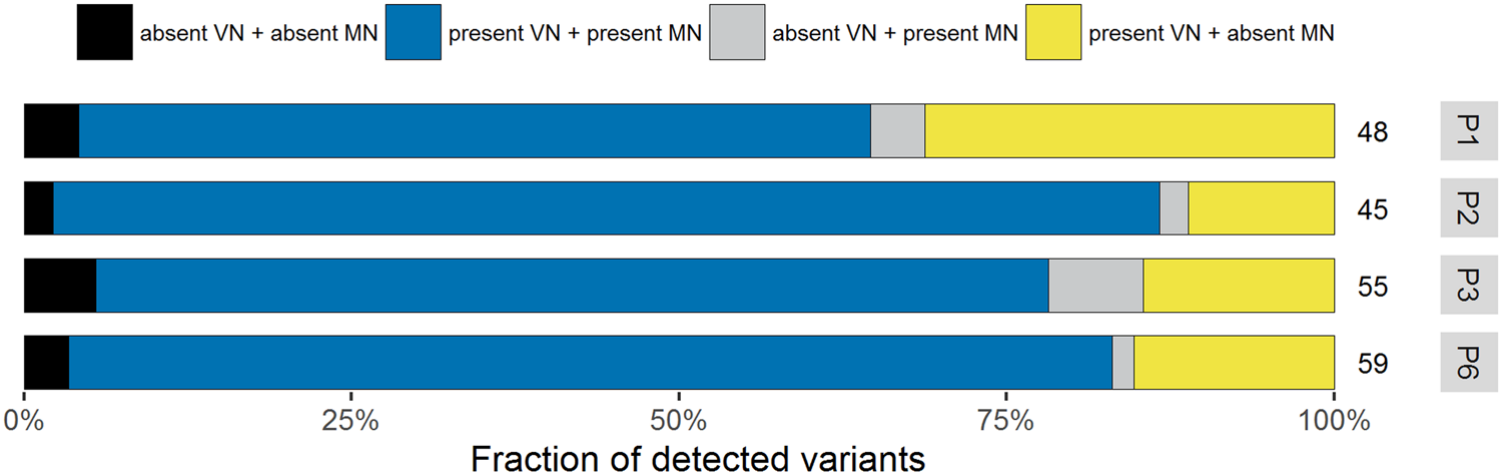
Defining somatic origin of variants using matched normal DNA (MN) or virtual normal genomes (VN) as reference. Annotation of variants detected in the primary tumors of the four patients of who matched normal was accessible (P1, P2, P3 and P6) are depicted relative to the total variants in that sample. In here, variants absent from both the VN and MN in black, variants present in both VN and MN in blue, variants absent in the VN but present in the MN in grey, and variants present in the VN but absent from the MN in yellow. The total number of detected variants is depicted at the right end of the bars.

Next, we aimed to define the somatic or germline origin of variants detected in the primary tumor material of the ten patients. Alignment to the reference genome revealed a total of 486 alternative variants in all ten primary tumors with a median of 50 variants (IQR 45–54 variants) per individual tumor. The subsequent identification of somatically acquired variants in tumor material involves the exclusion of germline SNPs from the called variants. Conventionally, SNPs detected in matched normal DNA are used for this purpose (see above). Correction for germline SNPs reported in the curated matched normal DNA left us with 17, 6, 11 and 11 putative somatic variants in the primary tumor of P1, P2, P3 and P6, respectively (Table 1). However, no suitable matched normal DNA for germline SNP detection was available for the remaining six patients. As an alternative approach, we explored the *in silico* Virtual Normal methodology^5^. This methodology applies the contextual information of variants reported in the public domain by not only taking into account the surrounding reference sequence, but also neighboring variants. We compared the conventional method using matched normal DNA (MN) with the in silico method using Virtual Normal genomes (VN) for the four patients of which suitable matched normal material was sequenced (Fig. 2). The concordance in classification as germline SNPs i.e. present in both the MN and VN, or as somatic variants i.e. absent from both the MN and VN, is in total median 81% of the variants detected in the primary tumors (Fig. 2, black and blue). Absent from the MN but present in the VN were 15% of the variants (Fig. 2, yellow) and 3% of the variants were absent from the VN but present in the MN (Fig. 2, grey).

**Table 1:** Variant detection in primary tumor and serum-derived cfDNA specimens.

Patient	Specimen	Variants	Variants in MN	Variants in VN	Somatic variants	Confirmed somatic variants
P1	Primary tumor	48	31	44	2	1
	cfDNA (serum T1)	46	29	42	2	2
	cfDNA (serum T2)	53	29	46	5	2
	cfDNA (serum T3)	77	30	47	28	1
P2	Primary tumor	45	39	43	1	1
	cfDNA (serum T1)	49	40	45	3	1
	cfDNA (serum T2)	46	40	44	1	1
	cfDNA (serum T3)	47	39	44	2	1
P3	Primary tumor	55	44	48	3	3
	cfDNA (serum T1)	57	45	49	4	3
	cfDNA (serum T2)	56	43	48	4	3
	cfDNA (serum T3)	55	44	48	3	3
P4	Primary tumor	36	NA	34	2	1
	cfDNA (serum T1)	33	NA	32	1	1
	cfDNA (serum T2)	33	NA	32	1	1
	cfDNA (serum T3)	35	NA	34	1	1
P5	Primary tumor	52	NA	49	3	3
	cfDNA (serum T1)	50	NA	47	3	3
	cfDNA (serum T2)	51	NA	48	3	3
	cfDNA (serum T3)	51	NA	48	3	3
P6	Primary tumor	59	48	56	2	1
	cfDNA (serum T1)	58	49	56	1	1
	cfDNA (serum T2)	59	50	57	1	1
	cfDNA (serum T3)	58	49	56	1	1
P7	Primary tumor	45	NA	35	10	8
	cfDNA (serum T1)	45	NA	34	11	7
	cfDNA (serum T2)	50	NA	36	14	6
	cfDNA (serum T3)	43	NA	34	9	8
P8	Primary tumor	51	NA	45	6	3
	cfDNA (serum T1)	49	NA	45	4	4
	cfDNA (serum T2)	49	NA	45	4	4
	cfDNA (serum T3)	48	NA	45	3	3
P9	Primary tumor	54	NA	52	2	2
	cfDNA (serum T1)	54	NA	52	2	1
	cfDNA (serum T2)	55	NA	54	1	1
	cfDNA (serum T3)	70	NA	54	16	1
P10	Primary tumor	41	NA	39	2	1
	cfDNA (serum T1)	111	NA	42	69	0
	cfDNA (serum T2)	54	NA	40	14	0
	cfDNA (serum T3)	44	NA	39	5	1

Number of variants (columns) in each specimen for each of the ten patients (rows). The columns indicate 1) total variants detected, 2) variants in the indicated specimen also detected in the matched normal specimen (if available), 3) variants in the indicated specimen also present in at least one Virtual Normal genome, 4) variants classified as somatic variant (criteria in manuscript) and 5) somatic variants in the indicated specimen also confirmed in at least one additional patient-matched specimen.

Taken together, we defined putative somatic variants in tumor DNA as follows. If matched normal material was available, occurrence of a tumor variant in this MN identifies variants as germline SNP. Additionally, irrespective the availability of matched normal material, in silico annotation using the VN genomes classifies variants as SNP when present in at least one VN genome. Applying the above to all variants of the ten primary tumors left us with 33 putative somatic variants recurrent at 26 distinct locations in the genome. Per individual tumor, a median of 2 (IQR 2–3) putative somatic variants were detected (Table 1, Supplementary Table S1).

### Detection of Variants in Minute Material

**Figure 3:**
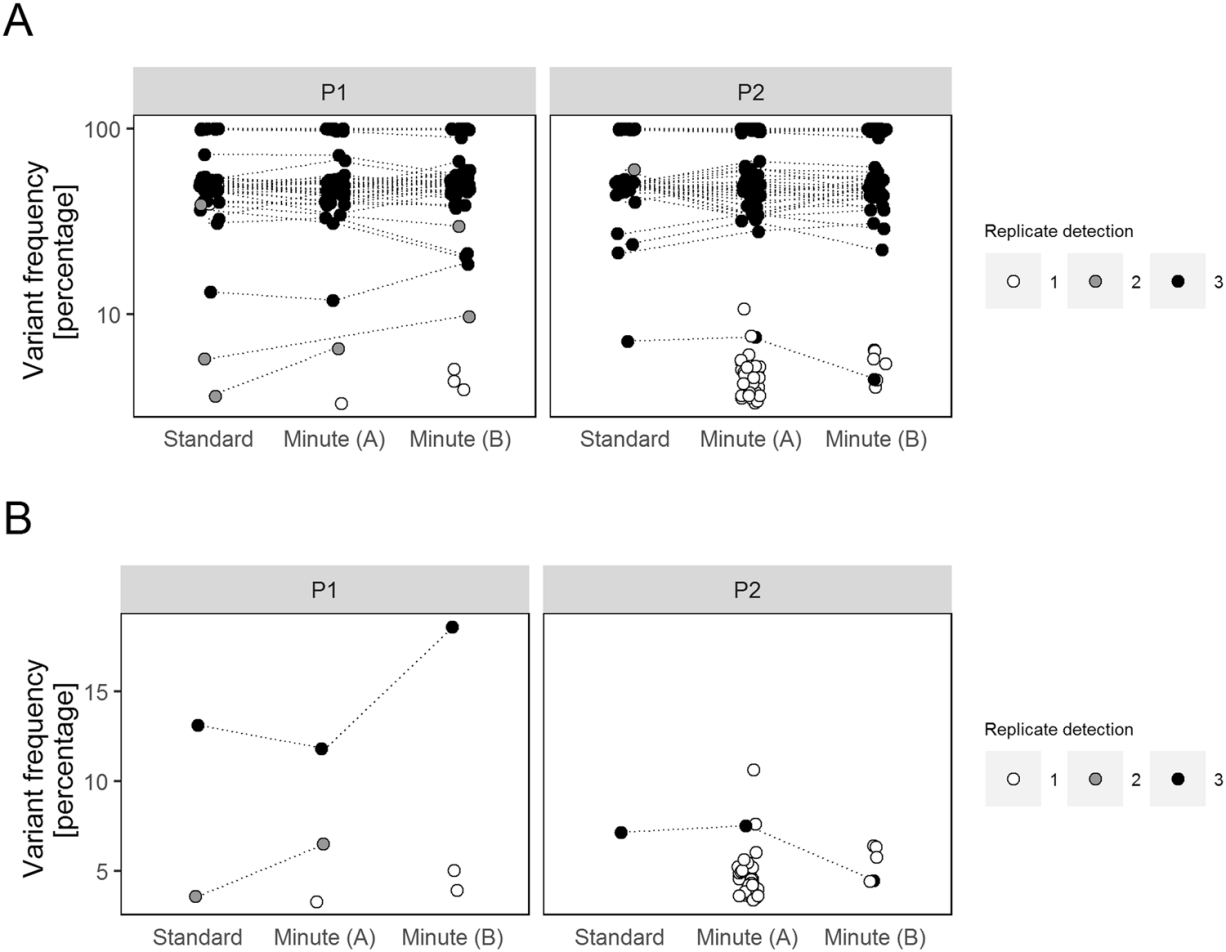
Detection of (somatic) variants in standard and minute primary tumor replicate samples. (**A**) The variant frequency in percentages of variants detected in a single replicate (1, white), in two out of three replicates (2, grey) or in all three replicates (3, black) of the two analyzed patients. Recurrent somatic variants at unique genomic positions are connected by lines to visualize variant frequency between replicates. (**B**) The variant frequency in percentages of somatic variants detected in a single replicate (1, white), in two out of three replicates (2, grey) or in all three replicates (3, black) of the two analyzed patients. Recurrent somatic variants at unique genomic positions are connected by lines to visualize variant frequency between replicates.

To evaluate the feasibility of detecting variants in minute quantities of DNA, we compared the sequence output of our targeted sequencing panel on primary tumor DNA as standard and minute sequencing input. To this end, we sequenced for two individual patients one standard as 10 ng input and two minute counterparts as 250 pg input. In the first patient, we detected a total of 48 variants in the primary tumor sequenced at standard amounts and 46 and 49 variants in the two replicates sequenced at minute amounts (P1, Table 2). In the second patient, we detected a total of 45 variants in the primary tumor sequenced at standard amounts and 79 and 50 variants in the two replicates sequenced at minute amounts (P2, Table 2). Using the variants detected in the standard primary tumor DNA as predicted positives, the variant detection in minute replicates has a sensitivity of 96.8% (IQR 3.1%) and a false discovery rate of 9% (IQR 14.4%). The high discovery rate implies false positive variants generated as a result of sequencing minute quantities. Indeed, a high number of variants is detected in only a single minute replicates sample and are present at a low variant frequency (Fig. 3A). To overcome these false positives, we aimed at the confirmation of a variant in an additional sample, which means we require a variant to be detected in at least two samples of an individual patient. A total of 44 variants were detected in both minute replicate samples for both P1 and P2. When we apply this confirmation-approach, a sensitivity of 91.7% (P1) and 97.8% (P2), and a false discovery rate of 0% (P1 and P2) were obtained. We next explored if the additional PCR cycles for the minute quantities introduced biases, such as a shift in variant frequency or allelic dropouts. For this, we selected variants in the primary tumor defined as germline by their presence in the VN genomes and with a variant frequency between 45–55% (heterozygotes, n = 46) or above 95% (homozygotes, n = 26). In the primary tumors sequenced at standard quantities, the median variant frequency is for heterozygotes 49.4% (IQR 3.57%) and for homozygotes 99.7% (IQR 0.74%). In the minute replicates, a larger variation is visible for those selected variants: for heterozygotes a median variant frequency of 49.2% (IQR 8.7%) (Brown-Forsythe Levene-type test P < 0.001) and for homozygotes 99.7% (IQR 1.5%) (Brown-Forsythe Levene-type test P = 0.08). However, we did not observe any allelic dropouts (a shift in variant frequency from 45–55% to <5% or >95%, or from >95% to <5%) (Supplementary Figure S3A).

**Table 2:** Variant detection in primary tumor specimen replicates at standard or minute input.

Patient	Replicate	Variants	Variants in MN	Variants in VN	Somatic variants	Somatic variants in 1/3	Somatic variants in 2/3	Somatic variants in 3/3
P1	Standard	48	31	44	2	0	1	1
	Minute (A)	46	29	41	3	1	1	1
	Minute (B)	49	30	44	3	2	0	1
P2	Standard	45	39	43	1	0	0	1
	Minute (A)	79	39	46	32	31	0	1
	Minute (B)	50	38	45	5	4	0	1

Number of variants detected (columns) in each replicate for each of the two patients of primary tumor specimen sequenced at standard and minute amounts (rows). The columns indicate 1) total variants detected, 2) variants in the indicated replicate also detected in the matched normal (MN) specimen (if available), 3) variants in the indicated replicate also present in at least one Virtual Normal (VN) genome, 4) variants classified as somatic variant (criteria in manuscript), 5) somatic variants in only the indicated replicate (one out of three) 6) somatic variants in the indicated and in one additional replicate (two out of three) and 7) somatic variants in the indicated and in all additional replicates (two out of three).

Last, for standard and minute replicates of the primary tumors, we defined the putative somatic variants for both patients (as described above). In the primary tumor DNA sequenced at standard input we detected a total of 2 and 1 somatic variants in P1 and P2, respectively. Of those variants, we validated in the minute replicates 2/2 (100%) (Minute A) or 1/2 (50%) (Minute B) somatic variants in P1 and 1/1 (100%) (Minute A) or 1/1 (100%) (Minute B) somatic variants in P2 (Fig. 3B). Thus, one of the three somatic variants was detected in one of the two minute replicates and therefore not detected by sequencing minute amounts.

Taken together, by sequencing only minute input quantities the majority of variants are detected however with a more variable variant frequency. The confirmation of variants in an additional patient-matched sample is necessary to minimize false discoveries, and this is therefore included in our workflow.

### Sequencing Minute cfDNA as Tumor Surrogate

**Figure 4:**
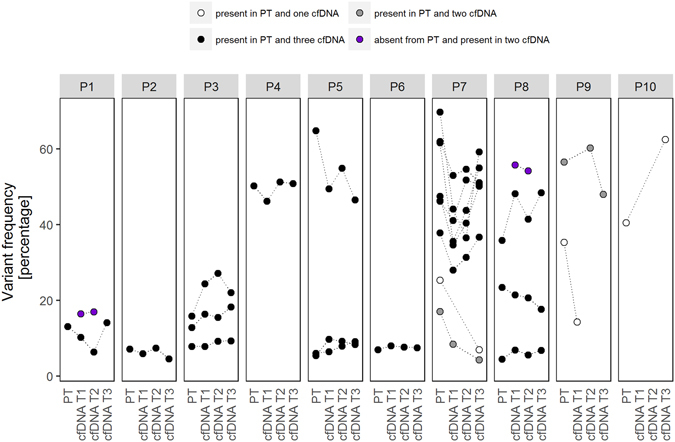
Detection of somatic variants in primary tumor and serum-derived cfDNA specimens. The variant frequency in percentages of somatic variants detected in the primary tumor (PT) and three consecutive cfDNA samples (either T1, T2 or T3) of the ten patients in the study. Recurrent somatic variants at unique genomic positions are connected by lines to visualize variant frequency between replicates. Somatic variants detected in the primary tumor and confirmed in one, two or three patient-matched cfDNA sample in respectively white, grey or black. Somatic variants absent from the primary tumor but detected in two cfDNA sample in purple.

Besides the primary tumors, we sequenced three consecutive serum-derived cfDNA samples at minute quantities for each of the ten patients, in which we defined the putative somatic variants (as described above). In the ten primary tumors we detected 33 putative somatic variants with median 2 (IQR 2–3) per individual, in the thirty sera we detected 219 putative somatic variants with median 3 (IQR 1–5) per cfDNA sample (Table 1). Because we included the confirmation of variants in an additional patient-matched sample, we first compared the putative somatic variants detected in each cfDNA sample with the matching primary tumor of each individual (Fig. 4). A total of 24 putative somatic variants were present in at least one cfDNA sample and the matching primary tumor. On the other hand, there were 2 putative somatic variants confirmed in multiple matching cfDNA samples of an individual that were absent from the matching primary tumor (Fig. 4, purple). Collectively, we were able to identify median 1 (IQR 1–3) putative somatic variants per cfDNA sample by confirmation in at least one additional patient-matched sample (Table 1). These 26 variants have potential as informative tumor-surrogate markers.

As a final step, we carefully inspected these variants to be able to use them as informative tumor surrogates (Table 3). First, we inspected the frequency of the variant. The contribution of non-tumor DNA is expected to be different between primary tumor specimen and cfDNA, and thus it would be unlikely for a somatic variant to have a cfDNA variant frequency similar to the variant frequency in the primary tumor specimen. Second, we employed public databases as *in silico* repositories for additional information about the detected variants, including databases on cancer driver genes^6^, variants in relation to human health^7^, germline polymorphisms^8–11^ and functional consequences of the observed substitution^12^. Third, to make sure the variant had not been filtered out in other patient-matched samples, we inspected the detected somatic variants in the original variant call files without any of the applied filtering steps of all the patient-matched samples. By using these three criteria, 22 variants are considered as non-informative (Table 3). The remaining four variants – *AKAP9* c. 1686T > G in P1, *PIK3CA* c.3140A > T and *SMAD4* c.1059C > A in P7, *TP53* c.520C > T in P9 –are considered informative tumor surrogates traceable as ctDNA by our targeted sequencing approach (Table 3). These four variants were validated by conventional Sanger sequencing, and/or independent re-sequencing (Supplementary Figure S4).

**Table 3:** Specifics of confirmed somatic variants in primary tumor and serum-derived cfDNA specimens.

Patient	Specimen	Gene	Variant	Variant frequency	Public databases							Filtering	Informative tumor surrogate
					COSMIC	ClinVar	GoNL	dbSNP	ESP	1000G	RefSeq		
P1	Primary tumor	*AKAP9*	c.1686T > G	no call							Missense	No	Yes
	cfDNA (serum T1)			16.5%									
	cfDNA (serum T2)			17.0%									
	cfDNA (serum T3)			no call									
	Primary tumor	*NCOR1*	c.540 G > C	13.1%							Missense	MN (8.4%)	No^†^
	cfDNA (serum T1)			10.3%									
	cfDNA (serum T2)			6.4%									
	cfDNA (serum T3)			14.2%									
P2	Primary tumor	*KMT2C*	c.1005T > A	7.1%	1 (other)			rs141993954	0.02		Silent	MN (6.5%)	No^†§^
	cfDNA (serum T1)			5.9%									
	cfDNA (serum T2)			7.4%									
	cfDNA (serum T3)			4.6%									
P3	Primary tumor	*KMT2C*	c.1005T > A	7.8%	1 (other)			rs141993954	0.02		Silent	MN (9%)	No^†§^
	cfDNA (serum T1)			7.8%									
	cfDNA (serum T2)			9.2%									
	cfDNA (serum T3)			9.3%									
	Primary tumor	*NCOR1*	c.540G > C	12.9%							Missense	MN (9.4%)	No^†^
	cfDNA (serum T1)			16.3%									
	cfDNA (serum T2)			15.5%									
	cfDNA (serum T3)			18.2%									
	Primary tumor	*PDE4DIP*	c.6933A > G	15.9%				rs3851872			Silent	MN (23.1%)	No^†§^
	cfDNA (serum T1)			24.4%									
	cfDNA (serum T2)			27.2%									
	cfDNA (serum T3)			22.1%									
P4	Primary tumor	*LRP2*	c.13685T > C	50.2%			0.01	rs142245618	0	0	Missense	MN (100%)	No^‡§^
	cfDNA (serum T1)			46.2%									
	cfDNA (serum T2)			51.3%									
	cfDNA (serum T3)			50.9%									
P5	Primary tumor	*AKAP9*	c.7541T > G	64.8%							Missense	MN (67.3%)	No^‡^
	cfDNA (serum T1)			49.5%									
	cfDNA (serum T2)			54.9%									
	cfDNA (serum T3)			46.5%									
	Primary tumor	*KMT2C*	c.1005T > A	6.0%	1 (other)			rs141993954	0.02		Silent	MN (9.2%)	No^†§^
	cfDNA (serum T1)			6.5%									
	cfDNA (serum T2)			7.9%									
	cfDNA (serum T3)			9.1%									
	Primary tumor	*NCOR1*	c.468A > G	5.5%							Silent	MN (6.2%)	No^†^
	cfDNA (serum T1)			9.7%									
	cfDNA (serum T2)			9.2%									
	cfDNA (serum T3)			8.4%									
P6	Primary tumor	*KMT2C*	c.1005T > A	6.9%	1 (other)			rs141993954	0.02		Silent	MN (6.4%)	No^†§^
	cfDNA (serum T1)			8.0%									
	cfDNA (serum T2)			7.7%									
	cfDNA (serum T3)			7.5%									
P7	Primary tumor	*APC*	c.7514G > A	47.5%			0	rs147549623	0		Missense	—	No^†§^
	cfDNA (serum T1)			34.7%									
	cfDNA (serum T2)			40.5%									
	cfDNA (serum T3)			50.1%									
	Primary tumor	*CDH1*	c.2336G > A	69.7%							Missense	—	No^‡^
	cfDNA (serum T1)			44.1%									
	cfDNA (serum T2)			36.5%									
	cfDNA (serum T3)			59.2%									
	Primary tumor	*KMT2C*	c.3384C > T	46.3%			0	rs144068847	0	0	Silent	—	No^‡§^
	cfDNA (serum T1)			41.1%									
	cfDNA (serum T2)			51.8%									
	cfDNA (serum T3)			50.6%									
	Primary tumor	*PDE4DIP*	c.6942T > C	37.9%				rs78461771			Silent	—	No^†§^
	cfDNA (serum T1)			28.0%									
	cfDNA (serum T2)			31.4%									
	cfDNA (serum T3)			36.7%									
	Primary tumor	*PIK3CA*	c.3140A > T	25.3%	>1 (breast)						Missense	S1 (2%)	Yes
	cfDNA (serum T1)			no call									
	cfDNA (serum T2)			no call									
	cfDNA (serum T3)			7.0%									
	Primary tumor	*RNF213*	c.10717G>A	61.6%							Missense	—	No^‡^
	cfDNA (serum T1)			35.6%									
	cfDNA (serum T2)			43.8%									
	cfDNA (serum T3)			55.0%									
	Primary tumor	*SMAD4*	c.1059C > A	17.0%							Nonsense	No	Yes
	cfDNA (serum T1)			8.5%									
	cfDNA (serum T2)			no call									
	cfDNA (serum T3)			4.3%									
	Primary tumor	*TP53*	c.639T > C	61.9%							Silent	—	No^‡^
	cfDNA (serum T1)			53.0%									
	cfDNA (serum T2)			54.6%									
	cfDNA (serum T3)			51.1%									
P8	Primary tumor	*APC*	c.7514G > A	35.8%			0	rs147549623	0		Missense	—	No^‡§^
	cfDNA (serum T1)			48.2%									
	cfDNA (serum T2)			41.4%									
	cfDNA (serum T3)			48.4%									
	Primary tumor	*KMT2C*	c.1005T > A	4.5%	1 (other)			rs141993954	0.02		Silent	—	No^†§^
	cfDNA (serum T1)			6.9%									
	cfDNA (serum T2)			5.6%									
	cfDNA (serum T3)			6.8%									
	Primary tumor	*NF1*	c.528T > A	no call	1 (other)	1	0.01	rs112306990	0	0	Missense	PT (34.4%) S3 (45.5%)	No^‡§^
	cfDNA (serum T1)			55.8%									
	cfDNA (serum T2)			54.2%									
	cfDNA (serum T3)			no call									
	Primary tumor	*PDE4DIP*	c.2997C > T	23.5%					0		Silent	—	No^†§^
	cfDNA (serum T1)			21.4%									
	cfDNA (serum T2)			20.6%									
	cfDNA (serum T3)			17.6%									
P9	Primary tumor	*ARID1A*	c.4120C > T	56.5%							Silent	S1 (49.6%)	No^‡^
	cfDNA (serum T1)			no call									
	cfDNA (serum T2)			60.3%									
	cfDNA (serum T3)			48.0%									
	Primary tumor	*TP53*	c.520C > T	35.4%	>1 (breast)						Nonsense	No	Yes
	cfDNA (serum T1)			14.3%									
	cfDNA (serum T2)			no call									
	cfDNA (serum T3)			no call									
P10	Primary tumor	*CREBBP*	c.2728A > G	40.5%			0	rs143247685	0	0	Missense	S1 (33.1%) S2 (35.2%)	No^‡§^
	cfDNA (serum T1)			no call									
	cfDNA (serum T2)			no call									
	cfDNA (serum T3)			62.5%									

Further inspection of the confirmed somatic variants in each specimen for each of the ten patients (rows). The columns indicate 1) targeted gene, 2) genomic location followed by the observed substitution as opposed to the reference genome, 3) in case of a call, variant frequency in indicated sample, 4) observation in public databases i.e. COSMIC, ClinVar, GoNL, dbSNP, ESP 1000G and RefSeq, 5) if the variant had been filtered out in patient-matched samples and if so, which samples with what variant frequency and 6) conclusion on the variants as an informative tumor surrogate with reasoning.

^†^Variant frequency similar in all patient-matched samples.

^‡^Variant frequency at heterozygosity in all patient-matched samples.

^§^Variant present in public polymorphism database.

## Discussion

We aimed at developing an easily implementable targeted NGS approach to detect tumor-specific somatic variants in only minute amounts of cfDNA, to apply this to retrospective cohorts with specimens collected over time. These cohorts often have restrictions, such as limited amounts available and suboptimal preservation of specimens. To have a comprehensive coverage of somatic variants, we designed an amplicon panel targeting exonic regions of 45 genes frequently mutated in breast, colon, prostate and ovarian cancer. Because of the small amplicon length in its design, this panel is suitable for the sequencing of fragmented DNA such as apoptotic cfDNA and degraded FFPE-derived DNA. Using this panel, we generated deep sequencing data of primary tumor DNA and minute amounts of serum-derived cfDNA samples of ten metastatic breast cancer patients from a retrospective cohort. We focused on the detection of single nucleotide substitutions in the sequencing data, because its detection is well-defined and it is the most prevalent mutation type in breast cancer^13^.

The only source of germline DNA was FFPE-preserved tumor specimen from which we were able to obtain adjacent healthy tissue for six of the ten patients. We observed a variable number of artefacts in these FFPE-derived DNA samples. False discovery of particularly C > T transitions is known for FFPE-derived DNA samples^14–17^, where cytosine residues are progressively de-aminated into uracil and upon amplification read as thymidine residues (C > U > T). This phenomenon is also evident in the GC content of the generated reads, where for the matched normal specimens a decrease in GC content (i.e. C > T transition) is observed (Supplementary Figure S1B). The specimens were collected between 25 and 37 years ago, which may partially explain the suboptimal quality of these samples. For the detection of germline SNPs, the detection threshold of at least 35% on variant frequency was sufficient to eliminate the majority of the preservation-induced artefacts such as the C > T substitutions in four of the six cases. When material is not intended to only call germline SNPs but also lower frequency variants, i.e. in FFPE preserved tumor specimens, alternative strategies to eliminate FFPE-induced deamination products prior to sequencing – such as enzymatic treatment of the DNA samples or the use of proof-reading polymerases – are likely required^15, 17, 18^.

As a next step, we aimed to define somatically acquired tumor-specific variants. The conventional approach uses matched normal DNA to make the distinction between somatic variants and germline SNPs in tumor material. However, when matched normal DNA is not of optimal quality – such as long-term FFPE preservation – the discrimination between germline SNPs and somatic variants becomes challenging. When artefacts are present in the matched normal data, there is a minor risk of erroneous categorization of tumor-specific variants as SNPs in the tumor material. Especially since the C > T transition is also often observed in somatic mutation profiles of cancer specimens^19^. Also, there is a major risk of classifying a germline SNP as somatic variant in tumor material when it is not detected in matched normal DNA. This seems to occur frequently for P1 (Fig. 2): the addition of an alternative approach to discriminate between somatic and germline variants by using VN genomes^5^ results in a large fraction of variants absent from the matched normal but present in the VN genomes. Also the fraction of variants present in both the matched normal and the VN genomes is small for P1. This indicates that some germline variants had been missed in the poor quality matched normal DNA. The *in silico* approach using VN genomes thus removed additional variants which had been missed by taking only the matched normal approach. By combining our matched normal material and VN genomes, we identified the putative somatic variants in the tumor genomes of the ten patients described here. As an additional control for our classification as germline or somatic variant, we reason that tumor cell content of primary tumor specimen or the tumor-derived cfDNA fraction is never 100%, and thus that somatic variants cannot display a variant frequency close to 100%. Indeed, none of the variants with a variant frequency above 95% were classified as somatic (Supplementary Figure S5A and S5B).

By sequencing only minute quantities, we observed in a few samples some unexpected high numbers of variants i.e. in a primary tumor sample sequenced at minute amounts (P2 Minute A, Table 2) and also a few serum-derived cfDNA samples sequenced at minute amounts (P1 serum T3, P9 serum T3, P10 serum T1, Table 1). Theoretically, polymerase introduced artefacts – with a mutation rate of high-fidelity polymerases in the order of one mutation per a million bases – can reach approximately 300 variants per targeted amplification (worst-case scenario with 21 PCR cycle amplification of a 139 bp amplicon). However, these randomly introduced artefacts should not exceed variant frequencies above 1.5% for minute quantities (a single artefact in 250 pg representing 36 diploid cells^20^) and 0.05% for standard quantities (a single artefact in 10 ng representing 1429 diploid cells), which is both below the 2% threshold used by the variant caller. However, we did observe a larger variation in variant frequency when using only minute input amounts for the primary tumor minute replicates (Supplementary Figure S3A) and also for the cfDNA samples (Supplementary Figure S3B). Amplification bias and variability in length of the fragmented DNA might skew the amplification of specific DNA fragments and thus variant frequency of detected variants^21^. We were unable to discover the exact reason why an increase in detected variants occurred in a few samples, and thus confirmation of detected variants is necessary. Commonly, validation of variants is achieved by independent re-analysis of the DNA sample. However, in retrospective cohorts this is not always possible due to restrictive sources of DNA. As an alternative, we suggest the confirmation of variants through the detection in at least two DNA samples of an individual patient. Because our cfDNA samples originate from consecutive serum draws, this means we might miss variants such as those occurring only during a specific stage in the disease trajectory. Also, we are aware that an obvious drawback of using only minute quantities of cfDNA is false negative detection. We can expect variants – especially low frequent variants – to be missed just by chance because of using minute fractions representing forty times fewer molecules than the standard input.

Thus, we were able to detect multiple putative somatic variants in primary tumor material as well as their corresponding serum-derived cfDNA samples. As a final step, we further inspected these variants whether they truly represent informative tumor surrogates. First, we compared variant frequency between patient-matched samples. The fraction of tumor-derived cfDNA is expected to be only a few percentages^22^, whereas the primary tumor specimens are expected to have high tumor cell content. Therefore, somatic variants at around 50% variant frequency in both the primary tumor as well as the cfDNA are likely heterozygous SNPs; somatic variants at a low variant frequency in both the primary tumor as well as the cfDNA are potential artefacts. Somatic variants with a clearly different variant frequency between the primary tumor and cfDNA, or between consecutive cfDNA draws at different disease stages (e.g. disease recurrence), are indicative for true somatic variants traceable as ctDNA. Second, we took an advantage of public databases on variants. However, we observe conflicting annotation, i.e. variants being reported as both germline SNP but also as a tumor-specific somatic mutation in public databases (Table 3). Thus, this information should be taken with caution. Third, we verified if – due to stringent threshold settings – the detected variants had not been filtered out in patient-matched samples. Based on these three aspects we defined if the variant detected in cfDNA are to be used as an informative tumor surrogate (Table 3).

A limitation by using only minute quantities of cfDNA is the detection limit in the order of 2% variant frequency (250 pg represents approximately 36 diploid cells^20^ thus allowing the detection of 1 mutant copy in 72 wildtype copies), whereas tumor-specific cfDNA can be present at variant frequencies as low as 0.001%^22^. Also, the retrospective serum samples used in this study are likely contaminated with DNA from non-tumor cells such as lysed leukocytes^23^, and thus the fraction of tumor-derived cfDNA is expected to be low. We are aware that the preservative in the blood collection tube (i.e. cloth activator or anti-coagulant), and processing procedure of the blood-derivative (i.e. time-to-processing and sedimentation speed), has a great influence on the cfDNA quantity^24–27^, and because this affects the fraction of tumor-derived cfDNA it should be kept in mind when analyzing cfDNA. Also, this makes comparison with other findings in literature quite complex. Previous studies have assessed cfDNA in metastatic breast cancer using methods varying from multi-gene to single mutation approaches including targeted or exome NGS, Sanger sequencing, digital (droplet) PCR, and BEAMing, most often in plasma samples. In comparison, our detection rate of 30% (3/10 patients with tumor-specific cfDNA) is at the low side compared to the reported detection rate of 50%-100% in studies with a similar approach (profiling multiple genes in both the primary tumor and the cfDNA^28–32^). We attribute this difference to the use of minute quantities and thus our ability to detect variants at or above 2% VAF, since in the above mentioned studies approximately 30% of the reported tumor-specific variants in metastatic breast cancer have a frequency below 2%. In addition, we designed our gene-panel for the most frequently mutated genes, but variants not included in the panel will not be detected.

Taken together, in a retrospective cohort with only limited amounts of serum-derived cfDNA available, using our developed workflow, we were able to retrace in cfDNA somatic variants detected in the primary tumor and also somatic variants not detected in the primary tumor for three of the ten patients (30%). We conclude that the presented approach enables specific detection of tumor-specific somatic variants above 2% variant frequency in minute amounts of cfDNA and can be used to discover tumor surrogate markers to explore the potential of cfDNA in oncology.

## Materials and Methods

### Specimens

Retrospectively collected specimens from metastatic breast cancer patients were obtained from our biobank. Selection was based on availability of fresh frozen primary tumor and serum samples at three different time points during treatment for metastatic disease and stored at −80 °C. The consecutive serum samples were collected at start of first line tamoxifen therapy (T1), during therapy (T2) and at disease progression (T3). For some cases haematoxylin-stained microscopic sections of the routine formalin-fixed and paraffin embedded primary tumor was available, of which macro-dissected adjacent normal epithelial mammary tissue yielded matched normal specimen^33^. The study was approved by the medical ethics committee (MEC 02.953) and performed according to the Code of Conduct of Medical Scientific Societies (www.federa.org/codes-conduct). In the Netherlands, according to the Code of Conduct, informed consent is not required for retrospective analysis of bio-specimens retrieved during standard of care procedures.

### DNA Extraction

Primary tumor DNA was extracted using the phenol chloroform method from pulverized FF specimens^34^. Matched normal DNA was extracted using Chelex 100 resin (Bio-Rad, Veenendaal, the Netherlands) as described previously^33^. CfDNA was extracted after external lysis of the serum (400 µL) using the automated MagNA Pure Compact Nucleic Acid Isolation Kit I (Roche Diagnostics, Almere, the Netherlands)^35^. Extracted DNA was quantified using the Qubit® 2.0 fluorometer (Thermo Fisher Scientific, Landsmeer, Netherlands).

### Amplicon-Based Targeted Next-Generation Sequencing

Ion semiconductor sequencing on the Ion Torrent Personal Genome Machine (PGM) was performed with an Ion AmpliSeq custom Panel applying consumables, kits, software packages and protocols of the manufacturer (Thermo Fisher Scientific). In short, adapter-ligated libraries were constructed using the AmpliSeq Library kit 2.0 using 3106 amplicons designed for small regions (63 to 139 nucleotides insert size) targeting 45 cancer-related genes^35^. Gene selection was based on the most frequently mutated driver genes in breast, colon, prostate and ovarian cancer as revealed by extensive genomic analysis deposited in the COSMIC database and interrogates for 39 genes all coding exons and for 6 genes only those exons harboring hotspot mutations (Supplementary Table S2), covering a total of 14159 COSMIC mutations. For sequencing of minute amounts, the recommended DNA starting input of 10 ng was reduced to approximately 250 pg (range 165.6–573.6 pg). For samples below 10 ng DNA input, adapter-ligated library preparation was adjusted from the standard 17 PCR cycles to 20 or 21 PCR cycles. After purification with AMPure XP beads (Beckman Coulter, Woerden, Netherlands), library quantity was determined using the Ion Library Quantitation kit and diluted to a final concentration of 8 pM. Template was emulsion PCR-prepared using the Ion PGM Template OT2 200 kit on the Ion OneTouch 2 system and confirmed using the Ion Sphere quality control kit. Template-positive Ion Sphere Particles were enriched using DynaBeads MyOne Streptavidin C1 on the Ion OneTouch ES instrument and barcoded samples were sequenced on the Ion Torrent PGM for 500 flows using the Ion PGM Sequencing Kit v2.0 on an Ion 318v2 chip.

### Bioinformatics and Statistics

Data from the PGM runs were processed initially using the Ion Torrent platform-specific pipeline software Torrent Suite to generate sequence reads, trim adapter sequences, filter, and remove poor signal-profile reads. Quality control of generated reads was performed using the Torrent Suite Software v4.0 with the “Coverage Analysis” plug-in and using FastQC^36^. Initial variant calling compared to the reference genome hg19 (build 37) was generated using Torrent Suite Software v4.0 with the “variant caller v4.0” plug-in. To be able to detect low frequency variants with minimal false negative calls we used the Somatic - Low Stringency Optimized settings. To correct for panel-specific sequencing errors, we removed all variants detected in >90% of the analyzed samples. Further analyses were conducted on single nucleotide variants in exonic regions only, an alternative variant read depth of at least 10x, a quality score of at least 20 and with a minimal strand bias threshold of 0.9. We applied to matched normal material – intended to detect germline SNPs at 50% or 100% variant frequency – a criterion of a at least 20x read depth. Other material – intended to detect tumor specific somatic variants – required a read depth of at least 100x. The Virtual Normal methodology was applied using the Galaxy tool in the DTLS tool shed, using the ‘Diversity and 1000G (479 genomes)’ virtual normal set. Annotation of the variants was performed by a custom pipeline including ANNOVAR^37^, the Catalogue Of Somatic Mutations In Cancer^6^ (COSMIC) version 67, ClinVar^7^, the Genome of the Netherlands^11^ (GoNL) version 7.0.0.59, Database of Single Nucleotide Polymorphisms^8^ (dbSNP) build ID 137 (non-flagged), 1000 Genomes^10^ (1000G) version 2012april_all, and the Exome Variant Server^9^ (ESP) version 6500si_all. Variants assigned as somatic were examined visually using Integrative Genomics Viewer (IGV) software. Statistical comparisons were performed by the indicated tests in R version 3.2.3.

## Electronic supplementary material

Supplementary Tables and Figures
