# Supplementary material for: Somatic Tumor Mutations Detected by Targeted Next Generation Sequencing in Minute Amounts of Serum-Derived Cell-Free DNA
Source: Sci Rep. 2017 May 18;7:2136. doi: 10.1038/s41598-017-02388-7 (PMC5437051; doi:10.1038/s41598-017-02388-7)

SUPPLEMENTARY INFORMATION FILE

SOMATIC TUMOR MUTATIONS DETECTED BY TARGETED NEXT GENERATION SEQUENCING IN MINUTE AMOUNTS OF SERUM-DERIVED CELL-FREE DNA

Marjolein J.A. Weerts[1]*, Ronald van Marion[2]*, Jean C.A. Helmijr[1], Corine M. Beaufort[1], Niels M.G. Krol[2,3], Anita M.A.C. Trapman-Jansen[1] , Winand N.M. Dinjens[2], Stefan Sleijfer[1], Maurice P.H.M. Jansen[1], John W.M. Martens[1]

[1]Erasmus MC Cancer Institute, Department of Medical Oncology and Cancer Genomics Netherlands, Rotterdam, The Netherlands

[2]Erasmus MC Cancer Institute, Department of Pathology, Rotterdam, The Netherlands

[3]Erasmus MC, Cancer Computational Biology Center, Rotterdam, The Netherlands

*Authors contributed equally

SUPPLEMENTARY TABLES

SUPPLEMENTARY TABLE S1 Specifics of putative somatic variants detected in primary tumor specimens.

Further inspection of the somatic variants in primary tumor specimens for each of the ten patients (rows). The columns indicate 1) targeted gene, 2) genomic location followed by the observed substitution as opposed to the reference genome, 3) variant frequency 4) observation in public databases i.e. COSMIC, ClinVar, GoNL, dbSNP, ESP 1000G and RefSeq, 5) if the variant is called in one or more of the consecutive cfDNA samples.

Patient	Gene	Variant	Variant frequency	Public databases							Detected in cfDNA
				COSMIC	ClinVar	GoNL	dbSNP	ESP	1000G	RefSeq	
P1	*NCOR1*	c.540G>C	13.1							Missense	Yes
	RNF213	c.14534C>A	3.6							Missense	No
P2	*KMT2C*	c.1005T>A	7.1	1 (other)			rs141993954	0.02		Silent	Yes
P3	*PDE4DIP*	c.6933A>G	15.9				rs3851872			Silent	Yes
	NCOR1	c.540G>C	12.9							Missense	Yes
	KMT2C	c.1005T>A	7.8	1 (other)			rs141993954	0.02		Silent	Yes

P4	*CDH1*	c.1596G>T	3.8						Missense	No
	LRP2	c.13685T>C	50.2		0.01	rs142245618	0.00	0.00	Missense	Yes
P5	*NCOR1*	c.468A>G	5.5						Silent	Yes
	KMT2C	c.1005T>A	6.0	1 (other)		rs141993954	0.02		Silent	Yes
	AKAP9	c.7541T>G	64.8						Missense	Yes
P6	*CREBBP*	c.3961G>T	22.9						Nonsense	No
	KMT2C	c.1005T>A	6.9	1 (other)		rs141993954	0.02		Silent	Yes
P7	*PDE4DIP*	c.6942T>C	37.9			rs78461771			Silent	Yes
	CDH1	c.2336G>A	69.7						Missense	Yes
	TP53	c.639T>C	61.9						Silent	Yes
	TP53	c.122T>G	34.8	>1 (other)					Missense	No
	RNF213	c.195G>A	5.8	1 (other)			0.00		Silent	No
	RNF213	c.10717G>A	61.6						Missense	Yes
	SMAD4	c.1059C>A	17.0						Nonsense	Yes

	PIK3CA	c.3140A>T	25.3	>1 (breast)					Missense	Yes
	APC	c.7514G>A	47.5		0.00	rs147549623	0.00		Missense	Yes
	KMT2C	c.3384C>T	46.4		0.00	rs144068847	0.00	0.00	Silent	Yes
P8	*PDE4DIP*	c.2997C>T	23.5				0.00		Silent	Yes
	ARID1A	c.4443C>A	3.8						Missense	No
	TP53	c.442A>G	34.7	>1 (other)					Missense	No
	LRP2	c.12948T>A	10.1						Silent	No
	APC	c.7514G>A	35.8		0.00	rs147549623	0.00		Missense	Yes
	KMT2C	c.1005T>A	4.5	1 (other)		rs141993954	0.02		Silent	Yes
P9	*ARID1A*	c.4120C>T	56.5						Silent	Yes
	TP53	c.520C>T	35.4	>1 (breast)					Nonsense	Yes
P10	*CREBBP*	c.2728A>G	40.5		0.00	rs143247685	0.00	0.00	Missense	Yes
	PIK3CA	c.3140A>T	47.9	>1 (breast)					Missense	No

SUPPLEMENTARY TABLE S2 Specifics of custom sequencing panel.

For each targeted gene (rows), the columns indicate 1) chromosomal position of the gene, 2) if the whole exonic region or a genomic hotspot region of the particular gene was targeted, 3) the start and end position in case of a hotspot region with column, 4) the number of exons targeted in case of whole exonic region and 5) the number of amplicons that amplify the indicated gene.

Gene	Chromosome	Target	Start-End *(position)*	Number of exons *(n)*	Amplicons *(n)*
AKAP9	chr7	ALL EXONS		51	157
AKT1	chr14	GENOME REGION	105246500-105246625		1
APC	chr5	ALL EXONS		16	97
ARID1A	chr1	ALL EXONS		21	74
ATM	chr11	ALL EXONS		62	138
BRAF	chr7	GENOME REGION	140453099-140453224		2
BRCA1	chr17	ALL EXONS		27	73
CDH1	chr16	ALL EXONS		16	35
CDK12	chr17	ALL EXONS		15	52
CHEK2	chr22	ALL EXONS		15	28
CREBBP	chr16	ALL EXONS		31	89
CTNNB1	chr3	GENOME REGION	41266061-41266175		2
EGFR	chr7	GENOME REGION	55241596-55241779<br>55259397-55259581		4
ERBB2	chr17	ALL EXONS		28	53

FBXW7	chr4	ALL EXONS		13	35
GATA3	chr10	ALL EXONS		6	16
JAK1	chr1	ALL EXONS		24	49
KAT6B	chr10	ALL EXONS		16	70
KIT	chr4	ALL EXONS		22	42
KRAS	chr12	GENOME REGION	25398183-25398310<br>25380240-25380323		3
LRP2	chr2	ALL EXONS		79	193
MAP2K4	chr17	ALL EXONS		11	23
MAP3K1	chr5	ALL EXONS		20	56
MED12	chrX	ALL EXONS		45	88
MLH1	chr3	ALL EXONS		21	37
KMT2A	chr11	ALL EXONS		37	135
KMT2D	chr12	ALL EXONS		54	184
KMT2C	chr7	ALL EXONS		59	193
MLLT3	chr9	ALL EXONS		11	22
NCOA3	chr20	ALL EXONS		25	55
NCOR1	chr17	ALL EXONS		48	102
NCOR2	chr12	ALL EXONS		50	97
NF1	chr17	ALL EXONS		59	143
PDE4DIP	chr1	ALL EXONS		56	120
PIK3CA	chr3	GENOME REGION	178952001-178952103<br>178936059-178936176		3

PIK3R1	chr5	ALL EXONS	18	33
PPP2R1A	chr19	ALL EXONS	15	27
PTCH1	chr9	ALL EXONS	26	57
PTEN	chr10	ALL EXONS	9	18
RB1	chr13	ALL EXONS	27	43
RNF213	chr17	ALL EXONS	69	185
RUNX1	chr21	ALL EXONS	10	21
RYR1	chr19	ALL EXONS	106	205
SMAD4	chr18	ALL EXONS	11	27
TP53	chr17	ALL EXONS	13	19

SUPPLEMENTARY FIGURE LEGENDS

SUPPLEMENTARY FIGURE S1 Read length distribution and per sequence GC content of the generated reads per sample type.

A: Density plot of read length distributions as calculated from the detected length of each read for each sample (black lines). The grey area indicates the expected read length distribution based on amplicon panel (grey area).

B: Density plot of per sequence GC content as calculated from the detected GC content of each read for each sample (black lines).

SUPPLEMENTARY FIGURE S2 Substitution specifics of variants called relative to the reference genome in DNA derived from matched normal and primary tumor specimens.

A: The variant frequency in percentages of variants detected in FFPE-derived matched normal specimens of six patients. Variants are color coded according to the six possible base substitutions (C > A in blue, C > G in black, C > T in red, T > A in grey, T > C in green and T > G in pink). The red dotted line indicates the 35% variant frequency threshold used to eliminate artefacts introduced by sample preservation.

B: Substitution spectra of variants called relative to the reference genome in DNA derived from fresh frozen (FF) primary tumor specimens. The contribution of the six possible base substitutions (as in A) are depicted for each of six relative to the total variants in that sample. The total number of detected variants is depicted at the end of the bars.

SUPPLEMENTARY FIGURE S3 Variant frequency of heterozygous and homozygous variants in standard and minute DNA input amounts.

A: Variant frequency of heterozygous (HET) and homozygous (HOM) variants for primary tumor DNA as standard input (black) or minute input (grey). Indicated are the median variant frequency with interquartile ranges and Brown-Forsythe Levene-type test for the equality of group variance P value.

B: Variant frequency of heterozygous (HET) and homozygous (HOM) variants for primary tumor DNA as standard input (black) or serum-derived cfDNA as minute input (grey). Indicated are the median variant frequency with interquartile ranges and Brown-Forsythe Levene-type test for the equality of group variance P value.

SUPPLEMENTARY FIGURE S4 Validation of the four variants considered informative tumor surrogates traceable as ctDNA by our targeted sequencing approach.

A: Sanger sequencing results of primary tumor and consecutive cfDNA samples of P1 in duplo, targeting the region of *AKAP9* containing variant c. 1686T>G. The amount of DNA used as input is indicated below the sample name. Signal for cytosine (C), adenine (A), thymine (T) and guanine (G) in respectively blue, green, red and black.

B: Re-sequencing results of variants *AKAP9* c. 1686T>G in P1, *PIK3CA* c.3140A>T and *SMAD4* c.1059C>A in P7, and *TP53* c.520C>T in P9. The variant frequency in percentages of the variants, where recurrent somatic variants at unique genomic positions are connected by lines to visualize variant frequency. Somatic variants detected in the primary tumor and confirmed in one or two patient-matched cfDNA sample in respectively white or grey. Somatic variants absent from the primary tumor but detected in two cfDNA sample in purple. Re-sequencing was performed as described by Jansen et al [1].

1. Jansen, M.P., et al., *Cell-free DNA mutations as biomarkers in breast cancer patients receiving tamoxifen.* Oncotarget, 2016. **7**(28): p. 43412-43418.

SUPPLEMENTARY FIGURE S5 Variant frequency of variants in primary tumor and serum-derived cfDNA specimens.

A: Histogram with the distribution of the variant frequency of the variants detected in the primary tumor specimens of ten patients classified as either germline (left, black) or somatic (right, grey). The red line indicates a variant frequency of 95%

B: Histogram as in A for the variants detected in the serum-derived cfDNA samples**.**

SUPPLEMENTARY FIGURE S1

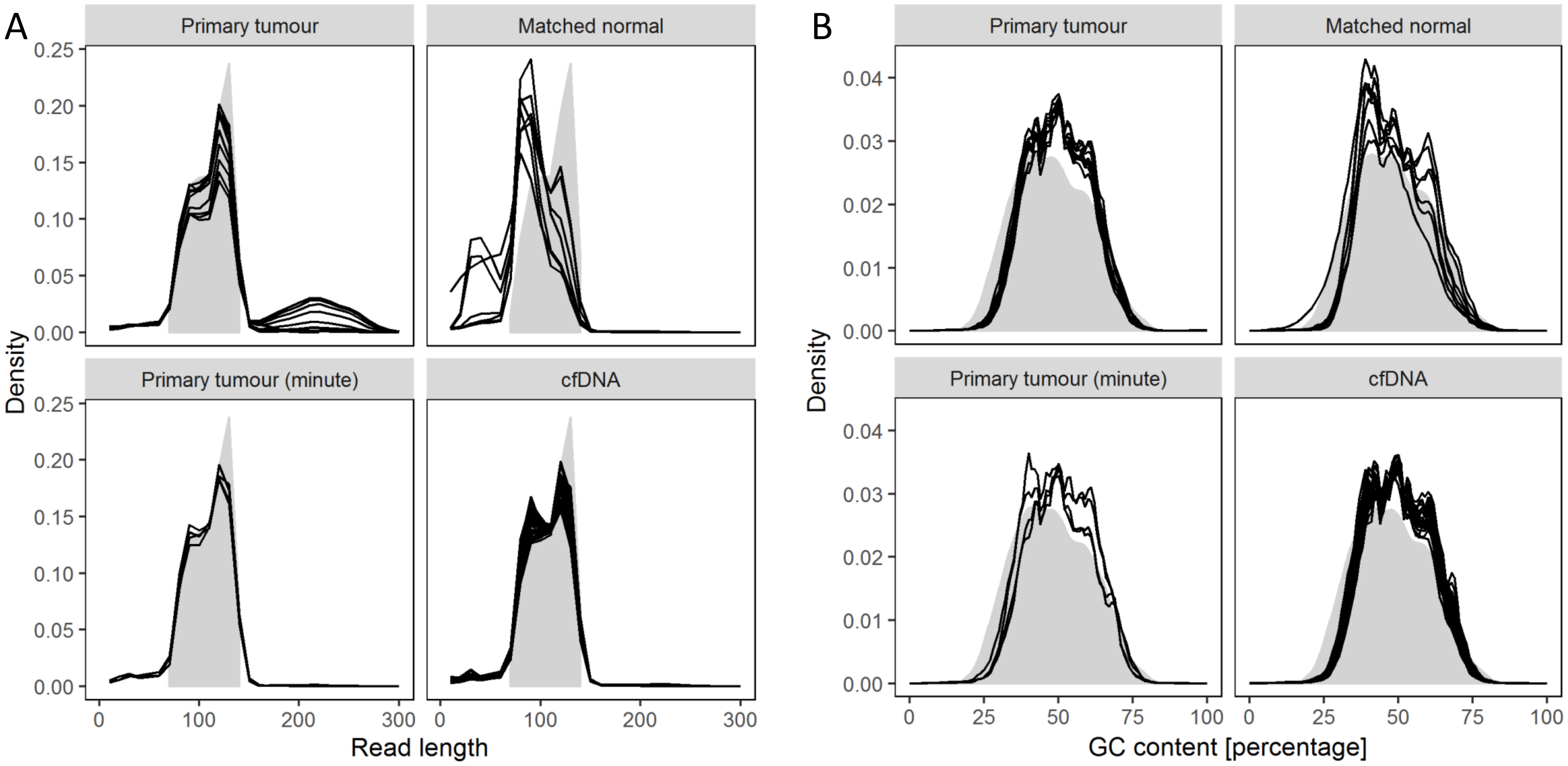

SUPPLEMENTARY FIGURE S2

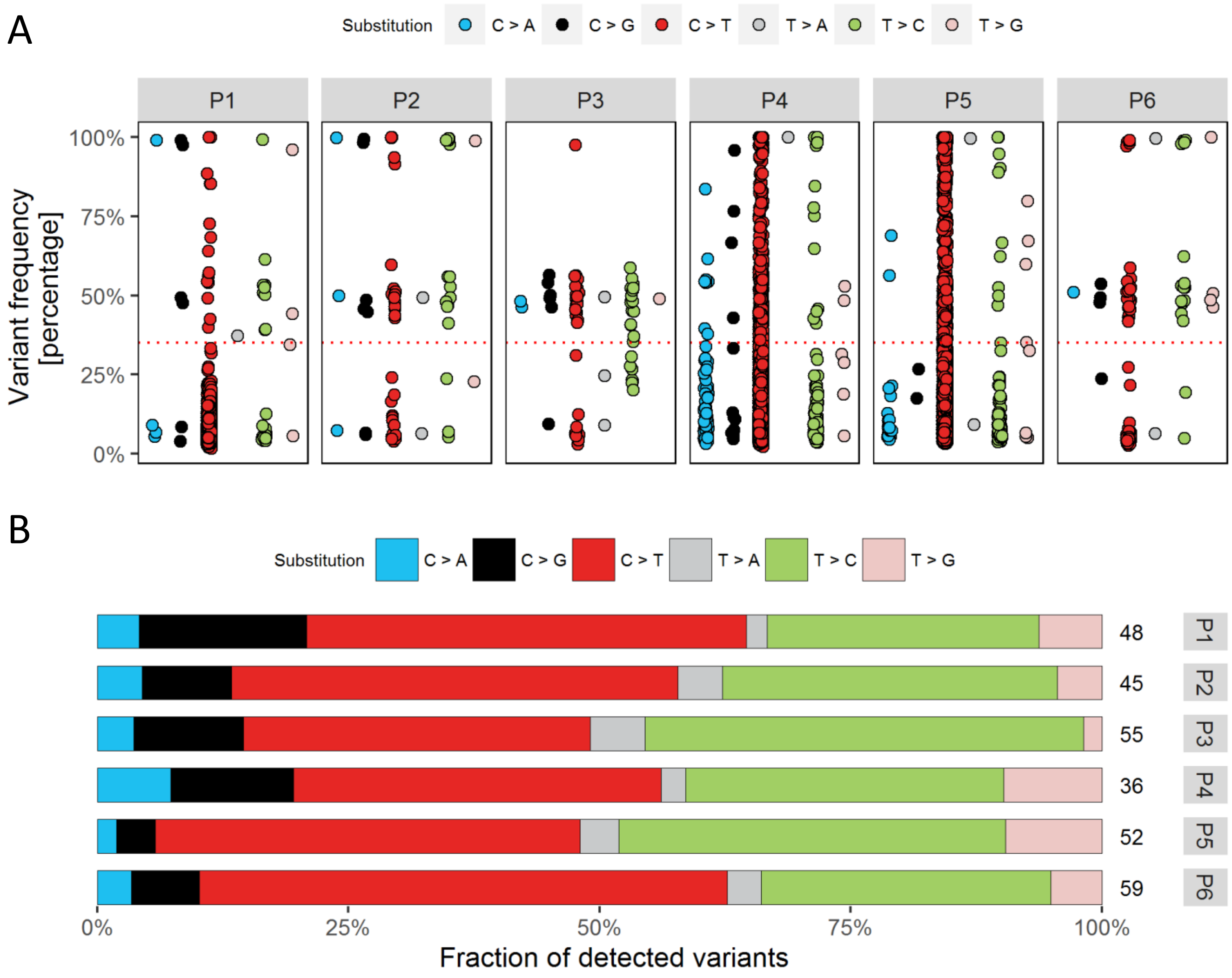

SUPPLEMENTARY FIGURE S3

A

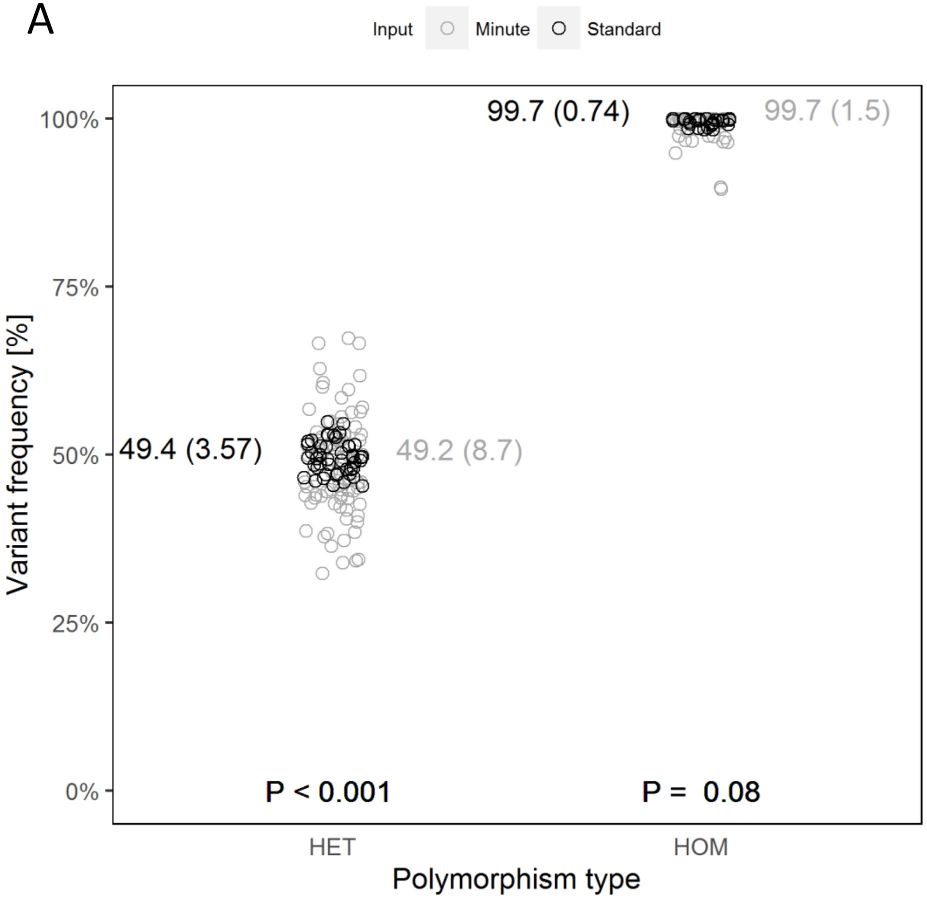

B

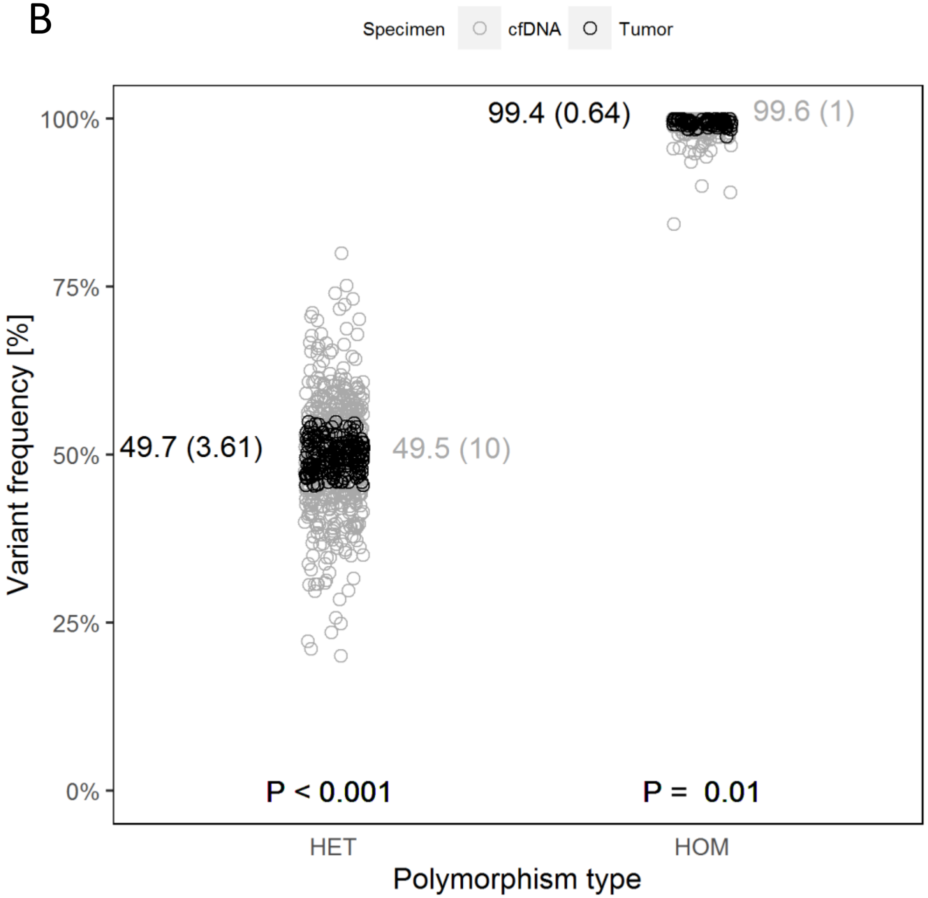

SUPPLEMENTARY FIGURE S4

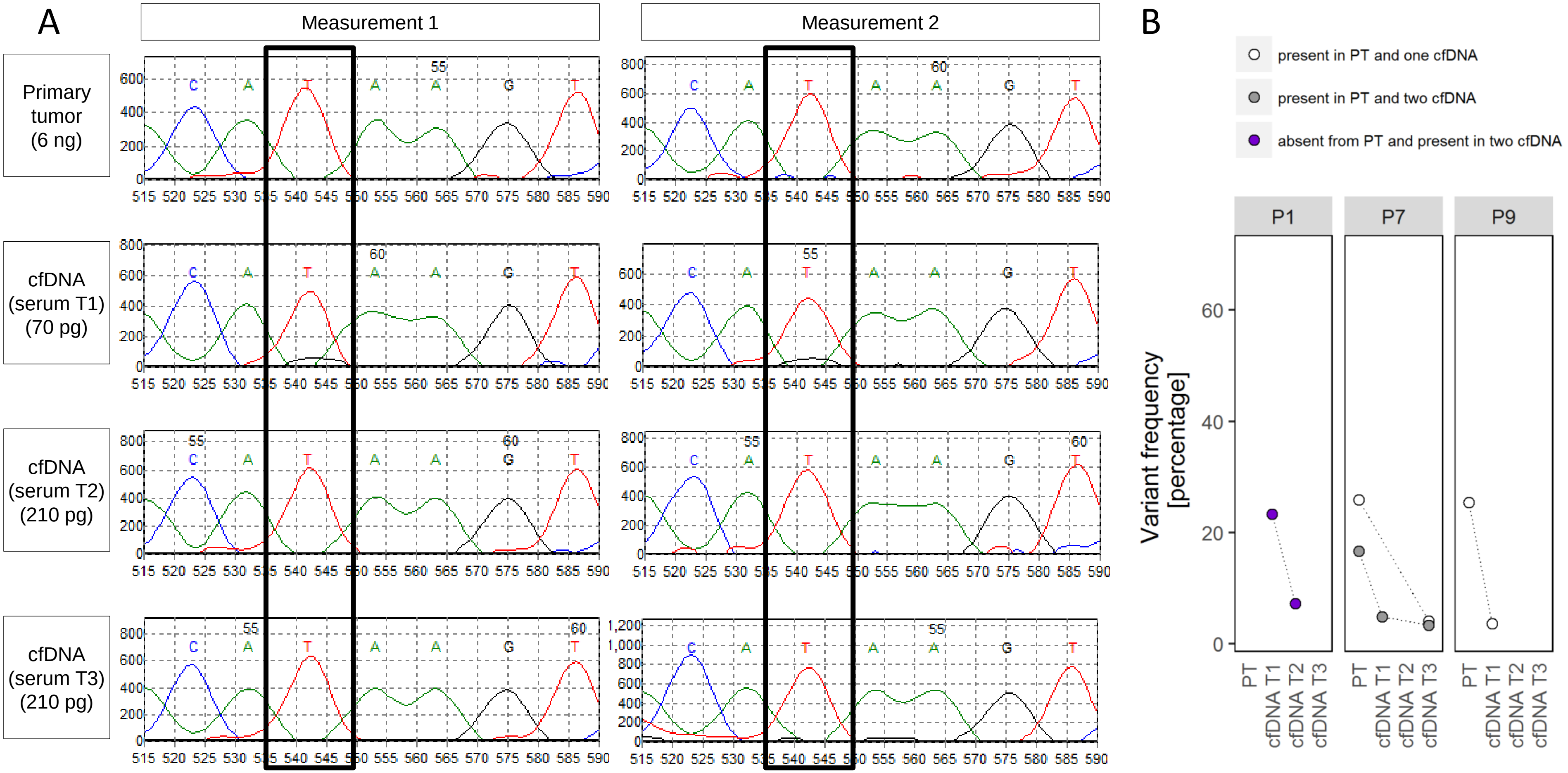

SUPPLEMENTARY FIGURE S5

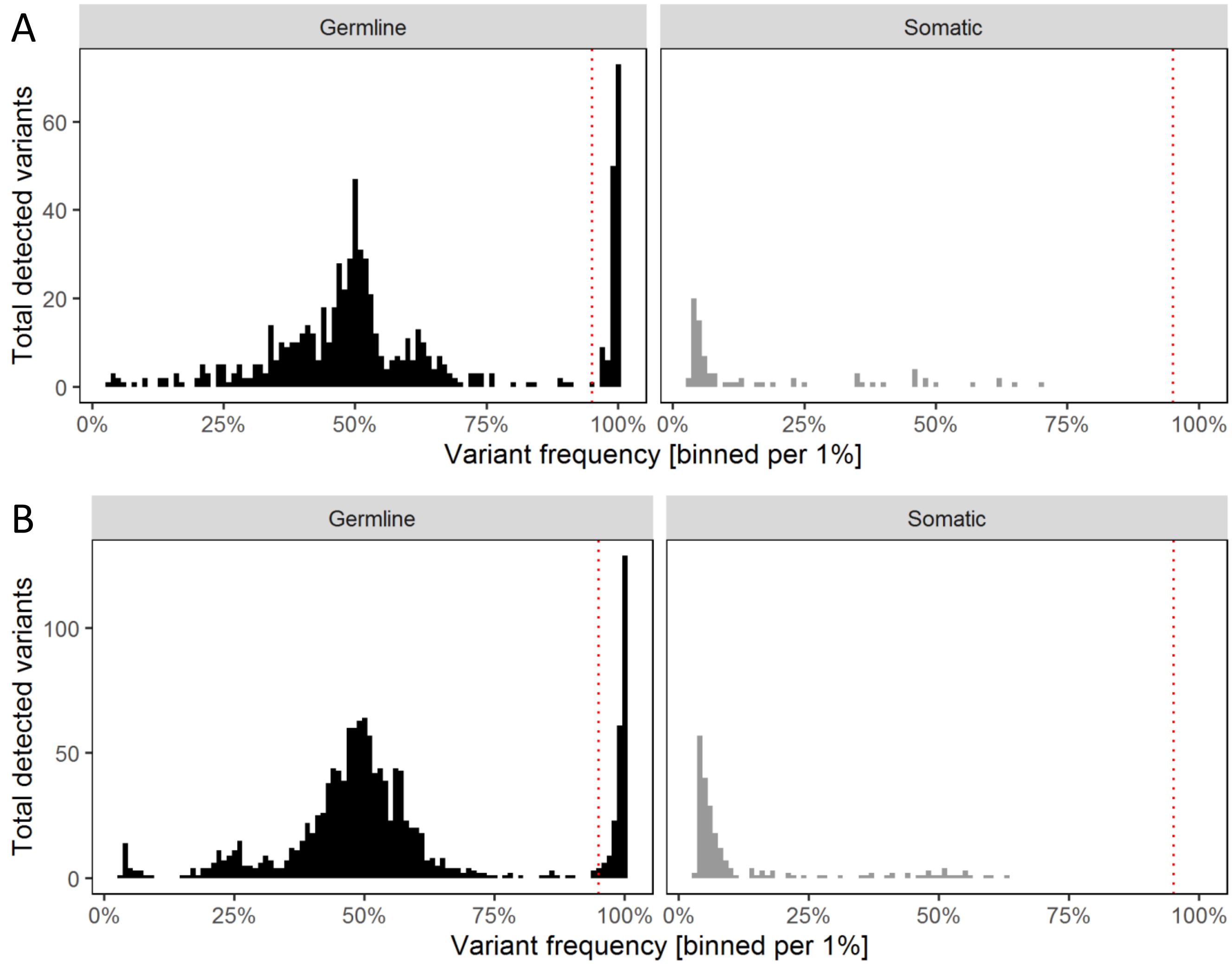

Supplement: Supplementary file 1 — Supplementary Tables and Figures [file 41598_2017_2388_MOESM1_ESM.pdf]
